# Breast cancer treatment and its impact on survival in Morocco: a study over a decade

**DOI:** 10.1186/s12885-024-12570-6

**Published:** 2024-07-01

**Authors:** Hind Mrabti, Catherine Sauvaget, Karima Bendahhou, Farida Selmouni, Richard Muwonge, Eric Lucas, Youssef Chami, Maria Bennani, Hassan Errihani, Abdellatif Benider, Rachid Bekkali, Partha Basu

**Affiliations:** 1 Institut National d’oncologie, CHU-Rabat, Rabat, Morocco; 2 Early Detection, Prevention and Infections Branch, International Agency for Research on Cancer, Lyon, France; 3 Cancer Registry of the Grand Casablanca Region, Casablanca, Morocco; 4 Lalla Salma Foundation - Prevention and Treatment of Cancers, Rabat, Morocco; 5 Centre Mohammed VI Pour Le Traitement Des Cancers, Centre Hospitalier Universitaire Ibn Rochd, Casablanca, Morocco

**Keywords:** Breast cancer, Management appropriateness, Determinants, Disease-free survival, Morocco

## Abstract

**Background:**

In Morocco, much progress has been made in breast cancer treatment. However, there is limited information on survival outcomes of breast cancer patients according to their therapeutic management.

**Methods:**

A pattern-of-care study was conducted in Morocco’s two main oncology centres: Rabat and Casablanca and has shown that major progress has been made in the quality of care with survival rates comparable to those in developed countries. The present study focuses on the different therapeutic strategies used in breast cancer and their impact on prognosis. Patients were classified into two categories: those considered as appropriately managed and those who were not.

**Results:**

A total of 1901 women with stage I to III breast cancer were included in this study, the majority (53%) were adequately managed and had better disease-free survival (DFS) rates than those who were not: DFS at 3 years (88% versus 62%) and at 5 years (80% versus 50%). Potential significant determinants of better management were: treatment in Rabat’s oncology centre, treatment between 2008 and 2012, being aged younger than 60 years, and early TN stage.

**Conclusion:**

This study demonstrated the value of proper integrated and coordinated management in a comprehensive cancer centre, to improve breast cancer survival.

**Supplementary Information:**

The online version contains supplementary material available at 10.1186/s12885-024-12570-6.

## Background

Breast cancer is the most common cancer in Morocco with incidence rates that have increased in recent years. Breast cancer accounts for 38.1% of all new cancer cases in women, with an age-standardized incidence rate of 45.6 per 100 000 women-years [1].

Treatment of breast cancer requires multidisciplinary management, including surgery, radiotherapy and systemic anti-cancer medications. Therapeutic strategies are complex and have evolved over time; they are adapted to the tumour grade, stage and molecular profile. Much progress has been made in breast cancer treatment: in addition to chemotherapy and endocrine therapy, the integration of targeted molecular therapies, in particular anti-HER 2 drugs, has revolutionized the management of Her2 positive breast cancer cases [2]. Moreover, therapeutic de-escalation in favour of endocrine therapy represents the current trend for luminal cancers with very good prognosis [3]. To preserve the patient’s quality of life, conservative treatment and the sentinel lymph node technique currently represent a standard of care when the characteristics of the tumour render it possible.

In Morocco, a lower-middle-income country in the eastern Mediterranean region, much progress has been made in the treatment of breast cancer, thanks to the collaboration between the Lalla Salma Foundation - Prevention and Treatment of Cancers and the Ministry of Health [4]. The main achievements have been the launch of a breast cancer early detection programme, the construction of two centres of excellence dedicated to the management of gynaecological and breast cancers, the establishment of a programme for vulnerable and indigent patients to access innovative drugs, including anti-Her2 therapies. At the same time, the Association Marocaine de Recherche et Formation en Oncologie Medicale published updated protocols for treatment of common cancers (5th edition) in September 2021 [5].

There is limited information from Morocco on survival outcomes of breast cancer patients according to stage and therapeutic management. We previously reported the socio-demographic characteristics and medical features of breast cancer patients as well as the factors associated with late presentation at the two largest oncology centres: the National Institute of Oncology in Rabat (INO), and the Centre Mohammed VI for cancer treatment in Casablanca (CM-VI) [6]. In this article, we report survival outcomes by stage and different therapeutic strategies based on a pattern-of-care study among women with breast cancer.

## Methods

This retrospective study included women with histologically confirmed breast cancer who were registered at INO and CM-VI between January 2008 and August 2017. A detailed study protocol and key outcomes were reported earlier [6]. A sampling of 2 months per year was used to include patients. Patients registered during the following periods were included: January-February 2008, March-April 2009, May-June 2010, July-August 2011, September-October 2012, November-December 2013, January-February 2014, March-April 2015, May-June 2016, July-August 2017.

Breast cancer patient files were collected from the medical records department of respective hospitals and data collection form was designed to extract information. The data collected included patients’ demographic information, clinical features (immunochemistry, TNM stage, grade, etc.), type of treatment received and compliance, as well as disease status at follow-up. Data collection was performed by trained doctoral students in Casablanca and research nurses in Rabat. Extracted information was verified by the principal investigator from each oncology centre and by the IARC coordinator. All data were entered in a dedicated online database.

Survival outcomes were examined according to different therapeutic strategies for localised or locally advanced breast cancer (stages I, II, and III only), treated with curative intent. For this analysis, patients were categorised into two groups: those considered as appropriately managed and those who were not. The definition of appropriate management was based on national and international recommendations in place during the study period, that was: all patients should have received surgery, all patients with breast-conserving surgery (BCS) should have received radiotherapy (RT), all patients with a node-involvement at pathology (pN) positive and/or a tumour size (pathology) > 5 cm (pT3/T4) should have received RT, all immunohistochemistry triple negative patients should have received chemotherapy (CT) (adjuvant and/or neoadjuvant), all human epidermal growth factor receptor 2 (HER2) positive patients with tumour size > 5 mm and/or pN positive should have received CT (adjuvant and/or neoadjuvant), all oestrogen receptor and/or progesterone receptor (ER/PR) positive should have received hormonotherapy, all oestrogen receptor and/or progesterone receptor (ER/PR) positive and HER2 negative patients with a tumour size > 2 cm (pathology) (pT2+) and/or a pN positive should have received CT (adjuvant and/or neoadjuvant), and all HER2 positive patients should have received trastuzumab [5, 7–10]. The patients who were managed differently were classified as inappropriately managed. Patients with partial treatment information were excluded from the main analyses.

Though the use of genomic signatures (such as Oncotype DX) has been included in international guidelines for breast cancer management since 2013 [8], given that genomic characterization of tumours is not widely available in Morocco (performed exceptionally in patients as out-of-pocket expenses), treatment informed by genomic characterization has not been included in the definition of appropriate management.

### Statistical analysis

Patient socio-demographic information, and women’s reproductive and tumour characteristics stratified by treatment appropriateness status were presented as proportions. The effect of these characteristics on treatment appropriateness status was assessed using Bayesian logistic regression models and presented as odds ratios together with their 95% credible intervals (CIs). Characteristics that were statistically significant in the multivariate logistic regression model were then adjusted for potential confounders in the disease relapse or recurrence outcome assessments. Vital status at last follow-up was reported as: alive and disease-free, alive with disease, alive with disease status unknown, dead, and status unknown. For the survival analysis, we used disease relapse or recurrence after treatment. The disease-free survival (DFS) endpoint was defined as being alive with disease (relapse) during the follow-up visit. DFS start date was the date of treatment initiation; while the end date was the date of relapse for the patients who met the endpoint criteria, or the date of death or the last visit date, whichever came first, for patients who did not meet the endpoint criteria. The impact of treatment appropriateness on DFS survival was assessed using Bayesian Cox proportional hazard regression models. The probability of relapse over the study duration, and at 3 and 5 years was estimated by Kaplan-Meier curves. Overall survival could not be assessed due to the insufficient number of deaths recorded in patients’ files to make the calculation. Due to missing information on outcomes, the data for years 2016 and 2017 were excluded from these DFS analyses.

The frequency of patient characteristics was assessed, and Kaplan Meier curves were developed in Stata 15.1 (StataCorp LP, Texas, USA), whereas the Bayesian regression models were run using Just Another Gibbs Sampler (JAGS) software [11, 12]. We also used JAGS to model the number of cases to complete missing information on the outcomes and/or explanatory variables [13].

This study was approved by the IARC Ethics Committee and the Ethics Committee of the Medical School, Rabat University.

## Results

A total of 1901 women with breast cancer detected at stages I to III were included in the analysis. 52% of women were aged < 50 years, 28% were aged 50–59 years and 19% were aged *≥* 60. Also, 49% of patients were premenopausal and 11% of patients had a family history of breast cancer. Patients predominantly came from urban areas (80%), were covered by the health insurance for indigent populations (47%), were married (78%), and half had three or more children. Cancer stage distribution showed 11% of patients at stage I, 45% at stage II and 36% at stage III (8% had missing stage information). Immunochemistry information was available for 79% of the patients; most tumours were ER and/or PR positive and HER2 negative (44%), followed by ER and/or PR positive and HER2 positive tumour type (17%), triple negative tumours (12%) and ER and PR negative/HER2 positive (7%). The main histopathology type was ductal carcinoma (78%). Most tumours were moderately differentiated (grade 2) (Supplemental Table 1).

Among 1755 breast cancer patients with stage and treatment information, 1412 (80%) received surgery (alone or in combination with RT and/or CT), and 1351 (77%) received chemotherapy (alone or within multimodality therapy). Multimodality therapy was used in 81% of patients. Taxanes were included in the chemotherapy protocol, in addition to anthracyclines (according to the AC60 or FEC 100 protocol), in 64% of cases. Among the 1250 women with oestrogen receptor and/or progesterone receptor (ER/PR) positive, 849 (68%) received hormonotherapy. Among the 415 cases with HER2 positive breast cancer, 149 (36%) received trastuzumab.

According to our definition of appropriate management, 53% of the patients were adequately managed. Table 1 reports the distribution of demographic, clinical and histological characteristics in patients appropriately managed. Statistically significant determinants of adequate therapeutic management included: being managed at INO, being managed before 2013, and presenting at an early stage. On the contrary, being aged > 60 years, and having an advanced stage were statistically associated with inadequate therapeutic management.

**Table 1:** Effect of socio-demographic, women reproductive and tumour characteristics on breast cancer patient appropriate treatment

	Patients	Patients appropriately				Crude analysis			Adjusted analysis*		
	assessed	treated				Odds ratio (95% CI)			Odds ratio (95% CI)		
	*n*	*n*	Proportion (95% CI)								
Overall	1719	919	0.5	(0.5 -	0.6)						
1. **Socio-demographic and women reproductive characteristics**											
Centre											
Casablanca	696	218	0.3	(0.3 -	0.3)	1.00			1.00		
Rabat	1023	701	0.7	(0.7 -	0.7)	4.84	(3.88 -	5.92)	6.07	(4.72 -	7.73)
Period											
2008–2012	725	471	0.6	(0.6 -	0.7)	1.00			1.00		
2013–2017	994	448	0.5	(0.4 -	0.5)	0.45	(0.36 -	0.54)	0.37	(0.27 -	0.49)
Age at diagnosis (years)											
< 40	304	170	0.6	(0.5 -	0.6)	1.00			1.00		
40–49	605	346	0.6	(0.5 -	0.6)	1.01	(0.75 -	1.33)	1.07	(0.73 -	1.46)
50–59	487	258	0.5	(0.5 -	0.6)	0.83	(0.60 -	1.11)	0.72	(0.45 -	1.07)
60–69	229	104	0.5	(0.4 -	0.5)	0.60	(0.39 -	0.83)	0.51	(0.29 -	0.81)
70+	94	41	0.4	(0.3 -	0.5)	0.62	(0.36 -	0.97)	0.44	(0.20 -	0.77)
Place of residence											
Urban	1379	737	0.5	(0.5 -	0.6)	1.00			1.00		
Semi-urban	146	76	0.5	(0.4 -	0.6)	0.95	(0.65 -	1.33)	1.21	(0.77 -	1.76)
Rural	194	106	0.5	(0.5 -	0.6)	1.07	(0.77 -	1.44)	1.24	(0.81 -	1.74)
Social security coverage											
None	573	330	0.6	(0.5 -	0.6)	1.00			1.00		
RAMED	862	431	0.5	(0.5 -	0.5)	0.74	(0.58 -	0.92)	1.14	(0.80 -	1.57)
CNOPS or CNSS	272	151	0.6	(0.5 -	0.6)	0.97	(0.69 -	1.31)	0.96	(0.59 -	1.38)
Marital status											
Never	271	140	0.5	(0.5 -	0.6)	1.00			1.00		
Ever	1442	777	0.5	(0.5 -	0.6)	1.04	(0.76 -	1.34)	1.24	(0.77 -	1.82)
Parity											
None	432	230	0.5	(0.5 -	0.6)	1.00			1.00		
1–2	433	230	0.5	(0.5 -	0.6)	0.89	(0.66 -	1.19)	0.75	(0.46 -	1.08)
3–4	455	240	0.5	(0.5 -	0.6)	0.84	(0.60 -	1.09)	0.75	(0.47 -	1.09)
5+	392	218	0.6	(0.5 -	0.6)	1.00	(0.72 -	1.32)	0.91	(0.57 -	1.36)
Menopausal status											
Pre	928	494	0.5	(0.5 -	0.6)	1.00			1.00		
Post	785	424	0.5	(0.5 -	0.6)	0.95	(0.76 -	1.14)	1.18	(0.84 -	1.62)
Family history of breast cancer											
No	1490	785	0.5	(0.5 -	0.6)	1.00			1.00		
Yes	222	133	0.6	(0.5 -	0.7)	1.40	(0.99 -	1.89)	1.42	(0.96 -	2.01)
2. **Tumour characteristics**											
Pathological T stage											
T1	378	207	0.5	(0.5 -	0.6)	1.00			1.00		
T2	987	565	0.6	(0.5 -	0.6)	1.14	(0.87 -	1.45)	1.04	(0.76 -	1.37)
T3	230	109	0.5	(0.4 -	0.5)	0.74	(0.51 -	1.01)	0.67	(0.41 -	0.96)
T4	116	38	0.3	(0.2 -	0.4)	0.40	(0.23 -	0.60)	0.35	(0.19 -	0.56)
Pathological N stage											
N0	763	424	0.6	(0.5 -	0.6)	1.00			1.00		
N1	487	257	0.5	(0.5 -	0.6)	0.89	(0.68 -	1.11)	0.83	(0.61 -	1.08)
N2	314	168	0.5	(0.5 -	0.6)	0.95	(0.71 -	1.24)	0.75	(0.52 -	1.02)
N3	148	70	0.5	(0.4 -	0.6)	0.72	(0.48 -	1.02)	0.60	(0.35 -	0.88)
Stage at diagnosis											
I	201	105	0.5	(0.5 -	0.6)	1.00			1.00		
II	855	503	0.6	(0.6 -	0.6)	1.30	(0.92 -	1.76)	1.22	(0.81 -	1.70)
III	657	311	0.5	(0.4 -	0.5)	0.82	(0.58 -	1.13)	0.62	(0.40 -	0.87)
Tumour type											
Ductal carcinoma	1475	830	0.6	(0.5 -	0.6)	1.00			1.00		
Lobular carcinoma	78	34	0.4	(0.3 -	0.6)	0.58	(0.33 -	0.88)	0.77	(0.40 -	1.28)
Others	162	52	0.3	(0.2 -	0.4)	0.40	(0.27 -	0.55)	0.41	(0.26 -	0.61)
Tumour differentiation											
Well	140	83	0.6	(0.5 -	0.7)	1.00			1.00		
Moderately	1001	505	0.5	(0.5 -	0.5)	0.76	(0.48 -	1.08)	1.02	(0.61 -	1.50)
Poorly	570	328	0.6	(0.5 -	0.6)	0.96	(0.60 -	1.39)	1.19	(0.68 -	1.79)
Molecular subtype											
ER and/or PR positive, and HER2 negative	932	508	0.5	(0.5 -	0.6)	1.00			1.00		
ER and/or PR positive, and HER2 positive	369	168	0.5	(0.4 -	0.5)	0.67	(0.51 -	0.87)	0.69	(0.48 -	0.92)
ER and PR negative, and HER2 positive	143	79	0.6	(0.5 -	0.6)	0.98	(0.64 -	1.40)	1.05	(0.62 -	1.58)
Triple negative	258	156	0.6	(0.5 -	0.7)	1.24	(0.89 -	1.69)	1.48	(0.99 -	2.13)

ER: Estrogen receptors; PR: Progesterone receptors; HER2: human epidermal growth factor receptor 2; * All appropriate patients characteristics included in the multivariate regression model

**Fig. 1:**
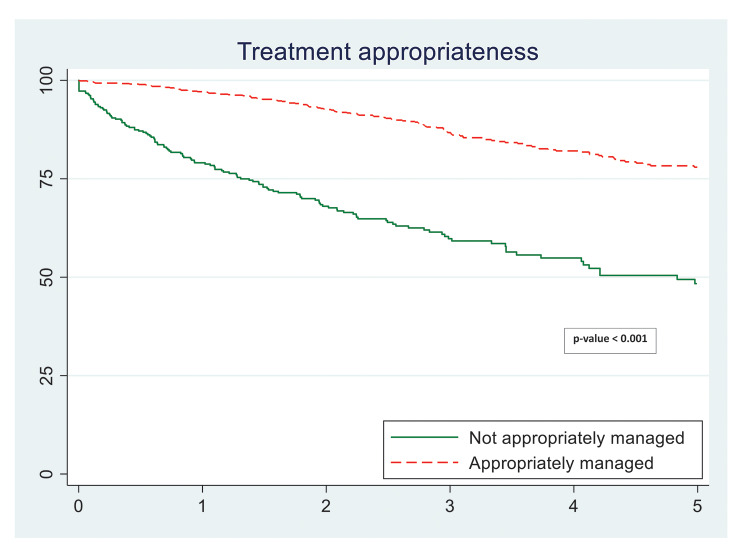
Kaplan Meier curves for survival to disease relapse among breast cancer patients according to treatment appropriateness

Table 2 shows that women treated appropriately had a statistically significant (65%) lower risk of breast cancer relapse during the mean 7-year follow-up period compared to those not managed appropriately. Figure 1 also shows significantly improved DFS in the appropriately managed group. Moreover, the appropriately managed patients had a better DFS at 3 years (88% versus 62%) and at 5 years (80% versus 50%) (Table 3).

**Table 2:** Effect of receipt of appropriate treatment on disease relapse among breast cancer patients in Morocco (2008–2015)

Receipt of appropriate treatment	Patients treated	Person-	Patients	Crude hazard rate (per 100 PYO)	Crude hazard ratio (95% CI)			Adjusted hazard ratio (95% CI)^a^		
		years of	with							
		observation	disease							
		(PYO)	relapse							
Not treated appropriately	436	1018.4	164	16.1	1.00			1.00		
Treated appropriately	794	2846.4	121	4.3	0.35	(0.26 -	0.45)	0.35	(0.26 -	0.45)

CI: confidence interval; PYO: person-years of observation; ^a^ Adjusted for period of registration, age at diagnosis, family history of breast cancer, pathological T stage, pathological N stage, tumour type, and clustering on centre due to the possible correlation of responses within centres

**Table 3:** Three- and 5-year survival to disease relapse among breast cancer patients in Morocco by receipt of appropriate management (2008–2015)

	Survival proportion (%)	
	3-year	5-year
	Overall	Overall
Receipt of appropriate treatment		
Not treated appropriately	61.8	49.8
Treated appropriately	87.8	79.5
Receipt of trastuzumab among HER2 positive patients		
Did not receive	69.2	62.6
Received	86.6	75.4
Receipt of trastuzumab and appropriate treatment among HER2 positive patients		
Did not receive trastuzumab and not treated appropriately	56.5	45.9
Received trastuzumab but not treated appropriately	74.0	42.3
Did not receive trastuzumab but otherwise treated appropriately	81.1	77.8
Received trastuzumab and treated appropriately	89.8	84.5
Taxane and treatment appropriateness among those eligible for chemotherapy		
Did not receive chemotherapy	79.1	79.1
Received chemotherapy other than taxane but not treated appropriately	55.5	42.2
Received taxane but not treated appropriately	61.8	40.1
Received chemotherapy other than taxane and treated appropriately	91.6	85.4
Received taxane and treated appropriately	86.3	73.4
Lumpectomy and mastectomy among patients who receive appropriate treatment		
Lumpectomy	91.8	83.0
Mastectomy	87.8	80.0

HER2: Human Epidermal Growth Factor Receptor 2

Table 3 reports the impact of trastuzumab or taxane use on DFS outcomes. There was a large difference in DFS between HER2 positive patients who received trastuzumab compared to those who did not (5-year DFS: 75% versus 63%). The benefit was clear even when we compared the well-managed HER2 positive patients receiving trastuzumab to those not receiving the drug. However, the benefits of using taxane among well-managed patients was not so evident. DFS were similar among patients who underwent mastectomy compared with those who had conserving treatment.

## Discussion

The results of this study are in line with data from a retrospective study conducted at INO in 2015: 88% of patients had undergone surgery. Chemotherapy was administered in 88% of patients, and 74% received radiotherapy. Half of patients received multimodality treatment. The proportion of patients receiving trastuzumab (13%) was lower than that found in the present study (36%) [14].

The proportion of patients receiving chemotherapy was high (77%) in the present study, while the proportion was 88% in the Mimouni study [14], and 66% in another study conducted in northern Morocco [15]. In a series of 2926 patients treated for stage I and II breast cancer, between 2013 and 2015, extracted from the American Surveillance, Epidemiology, and End Results (SEER) program database, chemotherapy was administered on average to 34% of cases in 2013 and to 21% in 2015 [16].

There is limited data on the therapeutic management of breast cancer in Morocco, as shown in a literature review published in 2014 [17]. All studies published to date on breast cancer in Morocco have focused on the epidemiological, clinical and molecular characteristics of breast cancer patients [18, 19].

This study reports on the impact that proper therapeutic management can have to decrease the risk of breast cancer relapse. Trastuzumab is a breakthrough treatment in the management of HER2 positive breast cancers. In Morocco, use of trastuzumab was associated with better survival. However, trastuzumab use must be integrated as part of a well-managed breast cancer care programme, alongside all other necessary treatments. This result is consistent with a systematic review where DFS was influenced by trastuzumab-containing regimens in women with early breast cancers [20]. We also found that HER2 positive patients were treated the least well, this is probably linked to the fact that only 36% of them received adjuvant trastuzumab.

Similarly, for chemotherapy-eligible cases, the adjunction of taxane must be integrated within a comprehensive and well-managed programme. Another systematic review reported that the use of taxane-containing adjuvant chemotherapy regimens improved DFS in women with operable early breast cancer, as compared to chemotherapy regimens without taxane [21].

Chemotherapy protocols in public oncology centres are based on the: “Guide to therapeutic protocols in oncology” which exists since 2011. This protocol is updated biannually; the latest version is dated September 2021 [5]. Protocols used for breast cancer are in accordance with international guidelines: if chemotherapy is indicated, a sequential protocol based on anthracyclines for 3 cycles followed by taxanes (weekly paclitaxel or docetaxel every 3 weeks) is recommended [22]. According to the present study, 36% of patients did not receive taxanes. This can be partly explained by missing records in the patient files but also by hospital pharmacies being out of stock, which could occur especially during the first period of the study.

Results showing that therapeutic management differed by oncology centre concur with survival results of this pattern-of-care study as management at INO was associated with better survival [6]. This may be explained by the fact that INO is a comprehensive cancer centre providing all cancer-related treatment services in one place, whereas in Casablanca’s oncology department is part of the university hospital which collaborates with departments that do not only take care of cancer patients specifically. Also in Casablanca, a large proportion of women initially have surgery immediately after the diagnosis of breast cancer at a different hospital (mostly in private clinics with a fee). They are then referred to CM-VI for chemotherapy and/or radiotherapy; treatment being free-of-charge since the cancer centre is a public facility. Nevertheless, we cannot draw any definitive conclusions due to lack of detailed patient characteristics from each centre.

Appropriate management was significantly more frequent during the first period 2008–2012. This can be explained by a chemotherapy de-escalation trend linked to a better selection of patients according to the molecular subtype. This is in accordance with international guidelines [8, 9, 22] which currently recommend chemotherapy only in high-risk breast cancer patients: namely triple negative, HER2 positive, majority of luminal B and exceptionally high-risk luminal A breast cancers. Moreover, appropriate management definition in this study did not take into account intermediate cases of luminal breast cancer where the choice between chemotherapy followed by endocrine therapy versus endocrine therapy alone is based on genomic signatures or at least a proliferative index like Ki67. A declining chemotherapy use trend was also found in the SEER program database: chemotherapy use decreased from 34.5 to 21.3% (from 26.6 to 14.1% for node-negative/ micrometastasis disease and from 81.1 to 64.2% for node-positive disease) [16].

This study has several limitations, especially due to its retrospective study design. Missing information on treatment appropriateness represented 19%, partly due to incomplete records, but also to patients lost to follow-up; the study protocol was based only on data collected from patient records, without the possibility of recalling patients for more information. Thus, the low number of patients receiving radiotherapy in the pattern-of-care study [6] could be due to the lack of data reported in patients’ medical records, and this is confirmed by the fact that in this study, DFS were similar between patients treated by mastectomy versus those who had conservative treatment.

The other limitation is the impact of classifying ER and/or PR positive invasive cancer patients without endocrine therapy information as inadequately managed. In a previous publication reporting the overall results of this cohort, 33 to 55% of ER and/or PR positive invasive cancer patients treated in Casablanca and 16 to 18% of ER and/or PR positive invasive cancer patients treated in Rabat did not receive endocrine therapy [6]. Since hormonotherapy is generally taken at home as an outpatient medication, this could explain the absence of information in the medical records. Therefore, it is likely that the percentage of patients receiving endocrine therapy was underestimated, especially in Casablanca, because endocrine therapy is a cheap and very accessible treatment in Morocco, and it is well tolerated by most patients. This underestimation of endocrine treatment has led to a better DFS among the inappropriately managed patients.

## Conclusion

This study demonstrated once again the value of managing breast cancer well in an integrated and coordinated comprehensive cancer centre. To improve patient survival, it is essential to have access to standardized therapies, but it is also crucial to have a well-organized pattern of care, permitting overall coordinated management within a multidisciplinary approach.

### Electronic supplementary material

Below is the link to the electronic supplementary material.

Supplementary Material 1

## Data Availability

The datasets used and/or analysed during the current study are available from the corresponding author on reasonable request.

## Abbreviations

BCS: Breast-conserving surgery
CM-VI: Centre Mohammed VI for cancer treatment in Casablanca
CT: Chemotherapy
DFS: Disease-free survival
ER/PR: Estrogen receptor and progesterone receptor
HER2: Human epidermal growth factor receptor 2
INO: National Institute of Oncology in Rabat
JAGS: Just Another Gibbs Sampler
pN: Node-involvement at pathology
RT: Radiotherapy
SEER: Surveillance, Epidemiology, and End Results registries
