# Supplementary material for: Breast cancer treatment and its impact on survival in Morocco: a study over a decade
Source: BMC Cancer. 2024 Jul 1;24:786. doi: 10.1186/s12885-024-12570-6 (PMC11218305; doi:10.1186/s12885-024-12570-6)
Supplement: Supplementary file 1 — Supplementary Material 1 [file 12885_2024_12570_MOESM1_ESM.docx]

**Supplementary Table 1: Distribution of socio-demographic, women reproductive and tumour characteristics of breast cancer patients in Morocco (2008-2017) by receipt of appropriate treatment**

|  | **Among patients with known** | | | | | **Among patients** | | **All patients** | |
| --- | --- | --- | --- | --- | --- | --- | --- | --- | --- |
|  | **treatment appropriateness status (n = 1719)** | | | | | **with unknown** | |  | |
|  | **Not appropriately** | | **Appropriately** | | **Chi^2^** | **treatment** | |  | |
|  | **treated** | | **treated** | | **p-value** | **appropriateness status** | |  | |
|  | **n (%)** | | **n (%)** | |  | **n (%)** | | **n (%)** | |
| Patients recorded | 800 |  | 919 |  |  | 182 |  | 1901 |  |
| Socio-demographic and women reproductive characteristics |  |  |  |  |  |  |  |  |  |
| Centre |  |  |  |  |  |  |  |  |  |
| Casablanca | 478 | (59.8) | 218 | (23.7) | <0.001 | 139 | (76.4) | 835 | (43.9) |
| Rabat | 322 | (40.3) | 701 | (76.3) |  | 43 | (23.6) | 1066 | (56.1) |
| Period |  |  |  |  |  |  |  |  |  |
| 2008-2012 | 254 | (31.8) | 471 | (51.3) | <0.001 | 58 | (31.9) | 783 | (41.2) |
| 2013-2017 | 546 | (68.3) | 448 | (48.7) |  | 124 | (68.1) | 1118 | (58.8) |
| Age at diagnosis (years) |  |  |  |  |  |  |  |  |  |
| <40 | 134 | (16.8) | 170 | (18.5) | 0.008 | 35 | (19.2) | 339 | (17.8) |
| 40-49 | 258 | (32.3) | 346 | (37.6) |  | 48 | (26.4) | 652 | (34.3) |
| 50-59 | 228 | (28.5) | 258 | (28.1) |  | 51 | (28.0) | 537 | (28.2) |
| 60-69 | 125 | (15.6) | 103 | (11.2) |  | 23 | (12.6) | 251 | (13.2) |
| 70+ | 52 | (6.5) | 41 | (4.5) |  | 24 | (13.2) | 117 | (6.2) |
| Missing | 3 | (0.4) | 1 | (0.1) |  | 1 | (0.5) | 5 | (0.3) |
| Place of residence |  |  |  |  |  |  |  |  |  |
| Urban | 642 | (80.3) | 737 | (80.2) | 0.894 | 135 | (74.2) | 1514 | (79.6) |
| Semi-urban | 70 | (8.8) | 76 | (8.3) |  | 18 | (9.9) | 164 | (8.6) |
| Rural | 88 | (11.0) | 106 | (11.5) |  | 29 | (15.9) | 223 | (11.7) |
| Social security coverage |  |  |  |  |  |  |  |  |  |
| None | 204 | (25.5) | 277 | (30.1) | 0.024 | 45 | (24.7) | 526 | (27.7) |
| RAMED | 398 | (49.8) | 400 | (43.5) |  | 96 | (52.7) | 894 | (47.0) |
| CNOPS or CNSS | 108 | (13.5) | 137 | (14.9) |  | 18 | (9.9) | 263 | (13.8) |
| Missing | 90 | (11.3) | 105 | (11.4) |  | 23 | (12.6) | 218 | (11.5) |
| Marital status |  |  |  |  |  |  |  |  |  |
| Never | 120 | (15.0) | 136 | (14.8) | 0.532 | 26 | (14.3) | 282 | (14.8) |
| Ever | 606 | (75.8) | 748 | (81.4) |  | 136 | (74.7) | 1490 | (78.4) |
| Missing | 74 | (9.3) | 35 | (3.8) |  | 20 | (11.0) | 129 | (6.8) |
| Parity |  |  |  |  |  |  |  |  |  |
| None | 159 | (19.9) | 212 | (23.1) | 0.662 | 29 | (15.9) | 400 | (21.0) |
| 1-2 | 180 | (22.5) | 218 | (23.7) |  | 41 | (22.5) | 439 | (23.1) |
| 3-4 | 195 | (24.4) | 226 | (24.6) |  | 46 | (25.3) | 467 | (24.6) |
| 5+ | 148 | (18.5) | 200 | (21.8) |  | 39 | (21.4) | 387 | (20.4) |
| Missing | 118 | (14.8) | 63 | (6.9) |  | 27 | (14.8) | 208 | (10.9) |
| Menopausal status |  |  |  |  |  |  |  |  |  |
| Pre | 375 | (46.9) | 477 | (51.9) | 0.952 | 78 | (42.9) | 930 | (48.9) |
| Post | 318 | (39.8) | 402 | (43.7) |  | 65 | (35.7) | 785 | (41.3) |
| Missing | 107 | (13.4) | 40 | (4.4) |  | 39 | (21.4) | 186 | (9.8) |
| Family history of breast cancer |  |  |  |  |  |  |  |  |  |
| No | 606 | (75.8) | 746 | (81.2) | 0.037 | 124 | (68.1) | 1476 | (77.6) |
| Yes | 74 | (9.3) | 126 | (13.7) |  | 14 | (7.7) | 214 | (11.3) |
| Missing | 120 | (15.0) | 47 | (5.1) |  | 44 | (24.2) | 211 | (11.1) |
| Tumour characteristics |  |  |  |  |  |  |  |  |  |
| Pathological T stage |  |  |  |  |  |  |  |  |  |
| T1 | 146 | (18.3) | 201 | (21.9) | <0.001 | 18 | (9.9) | 365 | (19.2) |
| T2 | 352 | (44.0) | 556 | (60.5) |  | 48 | (26.4) | 956 | (50.3) |
| T3 | 105 | (13.1) | 107 | (11.6) |  | 14 | (7.7) | 226 | (11.9) |
| T4 | 70 | (8.8) | 38 | (4.1) |  | 21 | (11.5) | 129 | (6.8) |
| Missing | 127 | (15.9) | 17 | (1.8) |  | 81 | (44.5) | 225 | (11.8) |
| Pathological N stage |  |  |  |  |  |  |  |  |  |
| N0 | 283 | (35.4) | 418 | (45.5) | 0.334 | 54 | (29.7) | 755 | (39.7) |
| N1 | 194 | (24.3) | 254 | (27.6) |  | 21 | (11.5) | 469 | (24.7) |
| N2 | 120 | (15.0) | 168 | (18.3) |  | 16 | (8.8) | 304 | (16.0) |
| N3 | 65 | (8.1) | 69 | (7.5) |  | 9 | (4.9) | 143 | (7.5) |
| Missing | 138 | (17.3) | 10 | (1.1) |  | 82 | (45.1) | 230 | (12.1) |
| Stage at diagnosis |  |  |  |  |  |  |  |  |  |
| I | 87 | (10.9) | 104 | (11.3) | <0.001 | 17 | (9.3) | 208 | (10.9) |
| II | 320 | (40.0) | 499 | (54.3) |  | 44 | (24.2) | 863 | (45.4) |
| III | 313 | (39.1) | 309 | (33.6) |  | 62 | (34.1) | 684 | (36.0) |
| Missing | 80 | (10.0) | 7 | (0.8) |  | 59 | (32.4) | 146 | (7.7) |
| Tumour type |  |  |  |  |  |  |  |  |  |
| Ductal carcinoma | 585 | (73.1) | 806 | (87.7) | <0.001 | 99 | (54.4) | 1490 | (78.4) |
| Lobular carcinoma | 41 | (5.1) | 33 | (3.6) |  | 12 | (6.6) | 86 | (4.5) |
| Others | 94 | (11.8) | 51 | (5.5) |  | 32 | (17.6) | 177 | (9.3) |
| Missing | 80 | (10.0) | 29 | (3.2) |  | 39 | (21.4) | 148 | (7.8) |
| Tumour differentiation |  |  |  |  |  |  |  |  |  |
| Well | 49 | (6.1) | 79 | (8.6) | 0.046 | 10 | (5.5) | 138 | (7.3) |
| Moderately | 410 | (51.3) | 486 | (52.9) |  | 76 | (41.8) | 972 | (51.1) |
| Poorly | 208 | (26.0) | 315 | (34.3) |  | 33 | (18.1) | 556 | (29.2) |
| Missing | 133 | (16.6) | 39 | (4.2) |  | 63 | (34.6) | 235 | (12.4) |
| Molecular subtype |  |  |  |  |  |  |  |  |  |
| ER and/or PR positive, and HER2 negative | 288 | (36.0) | 526 | (57.2) | 0.043 | 14 | (7.7) | 828 | (43.6) |
| ER and/or PR positive, and HER2 positive | 133 | (16.6) | 176 | (19.2) |  | 8 | (4.4) | 317 | (16.7) |
| ER and PR negative, and HER2 positive | 57 | (7.1) | 71 | (7.7) |  | 5 | (2.7) | 133 | (7.0) |
| Triple negative | 82 | (10.3) | 141 | (15.3) |  | 0 | (0.0) | 223 | (11.7) |
| Missing | 240 | (30.0) | 5 | (0.5) |  | 155 | (85.2) | 400 | (21.0) |
| ER: Estrogen receptors; PR: Progesterone receptors; HER2: human epidermal growth factor receptor 2 | | | | | | | | | |

**Supplement Table 2. Definition of appropriate management of breast cancer patients**

| **Condition** | **Appropriate Treatment** |
| --- | --- |
| All patients | Surgery |
| All breast-conserving surgery | Radiotherapy |
| All pN+ and/or pT3/T4 | Radiotherapy |
| All triple negative (ER- and PR- and HER2-) | Chemotherapy |
| All HER2+ with pT1b and/or pN+ | Chemotherapy |
| All ER/PR+ | Hormonotherapy |
| All HER2+ | Trastuzumab |
| All ER/PR+ and HER2- with pT2+ and/or pN+ | Chemotherapy |
